# The Formin Protein mDia2 Serves as a Marker of Spindle Pole Dynamics in Vitrified-Warmed Mouse Oocytes

**DOI:** 10.1371/journal.pone.0075729

**Published:** 2013-09-19

**Authors:** Hyejin Shin, Haengseok Song, Chang Suk Suh, Hyunjung Jade Lim

**Affiliations:** 1 Department of Biomedical Science & Technology, Institute of Biomedical Science & Technology, Konkuk University, Seoul, Korea; 2 Department of Biomedical Science, College of Life Science, CHA University, Seoul, Korea; 3 Department of Obstetrics and Gynecology, Seoul National University Bundang Hospital, Seongnam, Korea; State Key Laboratory of Reproductive Biology, Institute of Zoology, Chinese Academy of Sciences, China

## Abstract

The mouse diaphanous 2 (mDia2) protein belongs to the formin family and has been shown to nucleate actin filaments and stabilize microtubules, thus indicating a role in cytoskeleton organization. Our previous study, which showed that mDia2 specifically localizes to spindle poles of metaphase I mouse oocytes and NIH3T3 cells, provided the first evidence of its spindle pole-associated cellular function. In the present study, we aim to determine whether spindle pole proteins, such as mDia2 and pericentrin, can be used to monitor the status of spindle poles in cryopreserved mouse oocytes. We show herein that mDia2 exhibits an overlapping distribution with pericentrin, which is a crucial component of centrosomes and microtubule organizing centers (MTOCs). In vitrified-warmed oocytes, the overlapping distribution of mDia2 and pericentrin was immediately detected after thawing, thereby suggesting that mDia2 maintains a tight association with the spindle pole machinery. Interestingly, we observed that microtubules extend from mDia2 clusters in cytoplasmic MTOCs after thawing. This result suggests that mDia2 is a major MTOC component that is closely associated with pericentrin and that it plays a role in microtubule growth from MTOCs. Collectively, our results provide evidence that mDia2 is a novel marker of spindle pole dynamics before and after cryopreservation.

## Introduction

Formin proteins are known as actin nucleators [1]. Among them, Formin-2 (Fmn2) is associated with the formation of the actin cables required for spindle migration during oocyte maturation [2]–[4]. In addition, the absence of Fmn2 leads to a failed spindle migration and polar body extrusion in mice [5]. Mouse diaphanous 2, mDia2, is a closely related formin protein. mDia2 was first identified as a protein which interacted with small GTPases [6] and is involved in filopodia formation in mammalian cells [7], [8]. In mouse oocytes with no recognizable centrosomes [9], [10], microtubule organizing centers (MTOCs) serve the function of microtubule formation. MTOCs first appear as scattered small clusters in the ooplasm during prophase I [11]. These structures gather near the condensed chromosomes during prometaphase I and start to form spindle poles [10], [12]. Therefore, proteins generally known as centrosomal proteins, such as γ-tubulin and pericentrin, are localized in the MTOCs of oocytes [10], [13], [14]. Using mouse oocytes, we showed for the first time that mDia2 is exclusively localized to the spindle pole and co-localizes with γ-tubulin [15]. mDia2 also localizes to the mitotic spindle poles of NIH3T3 cells [15]. Thus, mDia2 appears to have multiple cellular functions that include filopodia formation and spindle pole organization, both of which involve dynamic establishment of microtubule. However, no investigations of mDia2 functions have been performed since its expression in mouse oocytes was initially observed.

Cryopreservation of oocytes is widely used to store oocytes of various species for future use [16]–[19]. Vitrification is a fast cooling process which is considered more convenient process of oocytes cryopreservation than slow freezing. While the survival rate of oocytes after vitrification and warming is quite high, the oocyte quality must be assessed at the cellular and molecular level to minimize the reduction of developmental competence after fertilization. Initially, noninvasive observations are conducted, such as an analysis of the overall morphology, discoloration, and spindle shape by using Polscope technology [20]–[24]. In addition, the effects of freezing on the meiotic spindle shape and length have been studied using various techniques [25]–[27]. However, the effects of vitrification on the formation of ring-shaped spindle poles and the localization of spindle pole proteins after freezing and thawing has not been investigated. Meiotic nondisjunction is the leading cause of chromosomal abnormalities in oocytes [28]. Importantly, a more detailed analysis that delves beyond the morphology of the spindle would allow insightful and detailed study of the meiotic maturation process. From a morphological standpoint, the meiotic spindle is a barrel-shaped form with γ-tubulin ring structures at each end [14]. Therefore, spindle poles in oocytes are wider than those of somatic cells undergoing mitosis. In mouse oocytes, mDia2 also possesses a wide ring-like morphology at each end and shows a complete overlap with γ-tubulin rings [15].

In the present study, we analyzed two aspects of mDia2. First, we assessed whether the distribution of mDia2 overlaps with that of pericentrin, which is a crucial component of centrosomes and MTOCs. Second, we investigated whether the study of mDia2 would be an effective strategy to monitor spindle pole dynamics in vitrified-warmed oocytes. We report herein that mDia2 exhibits an overlapping distribution with pericentrin before and after oocyte thawing, and functions as a member of MTOCs during vitrification and thawing.

## Results

### mDia2 co-localizes with pericentrin in mouse oocytes

**Figure 1:**
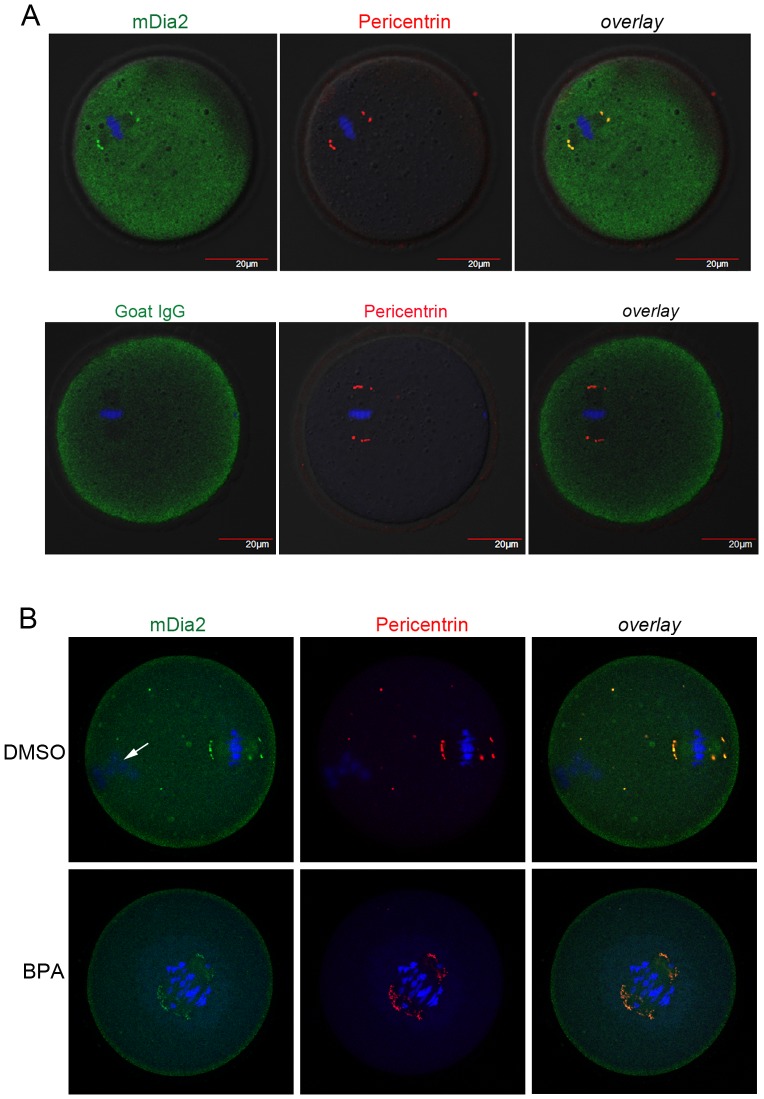
mDia2 is localized to spindle poles of MII oocytes. Immunofluorescence staining of mDia2 and pericentrin in mouse MII oocytes. Oocytes were stained with goat anti-mDia2 and mouse monoclonal anti-pericentrin antibodies. Overlapped distribution of mDia2 and pericentrin generates yellow fluorescence (overlay). Primary antibodies were used at 2 µg/ml. DNA is counter-stained with TO-PRO-3-iodide. Green, mDia2; red, pericentrin; blue, DNA.

We previously observed that in mouse oocytes, mDia2 localizes to the meiotic spindle pole and its distribution overlaps with that of γ-tubulin [15]. Pericentrin is a component of the pericentriolar materials and is used as a marker of centrosomes and MTOCs [13], [14], [29]. We herein examined whether the distribution of mDia2 overlaps with that of pericentrin in MII oocytes. By confocal microscopy, we observed that mDia2 and pericentrin exhibit co-localization on spindle poles (Fig. 1). This confirms that mDia2 is a novel component of spindle pole in mouse oocytes.

### Co-localization of mDia2 and pericentrin in vitrified-warmed mouse oocytes

**Figure 2:**
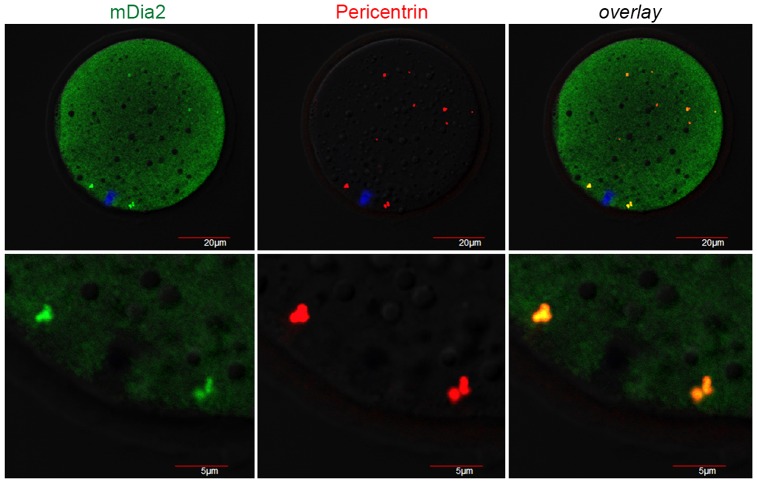
Localization of mDia2 and pericentrin in vitrified-warmed oocytes. MII oocytes were vitrified and stored in LN_2_ for 4 weeks. After thawing, oocytes were subjected to immunofluorescence staining with anti-mDia2 and anti-pericentrin antibodies. Overlapped distribution of mDia2 and pericentrin generates yellow fluorescence (overlay). DNA is counter-stained with TO-PRO-3-iodide. Green, mDia2; red, pericentrin; blue, DNA. The bottom panel shows the magnified region of the chromosome-spindle complex. DNA is not visible in high magnification images because it is out of focus.

**Figure 3:**
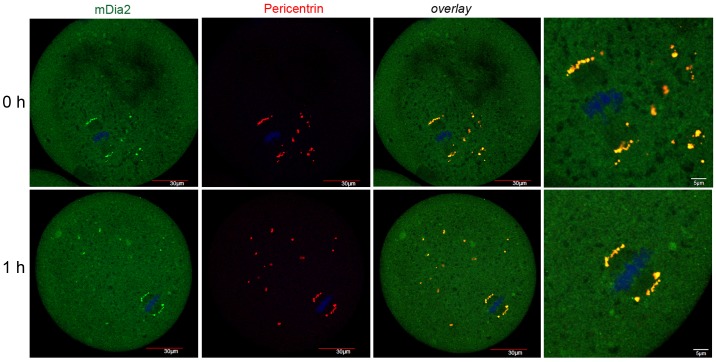
Localization of mDia2 and pericentrin at the time of thawing. Vitrified MII oocytes were stored in LN_2_ for 2 weeks. Oocytes were taken out from LN_2_, incubated in decreasing concentrations of sucrose, and then fixed immediately (0 h). Some oocytes were incubated in 37 C for recovery for 1 h after thawing (1 h). These oocytes were subjected to immunofluorescence staining with anti-mDia2 and anti-pericentrin antibodies. Overlapped distribution of mDia2 and pericentrin generates yellow fluorescence (overlay). DNA is counter-stained with TO-PRO-3-iodide. Green, mDia2; red, pericentrin; blue, DNA. The right panel shows the magnified region of the chromosome-spindle complex.

We next examined if the localization of mDia2 on spindle poles maintained in cryopreserved oocytes. Therefore, we performed double immunofluorescence staining of mDia2 and pericentrin in vitrified-warmed oocytes. Oocytes were stored in LN_2_ for 4 weeks and then thawed in decreasing concentrations of sucrose (see Materials and methods). As shown in Fig. 2, the vitrified-warmed oocytes after recovery exhibit overlapping distribution of mDia2 and pericentrin at the spindle poles. We also examined the time-based localization of these proteins during thawing and recovery. The vitrified oocytes were immediately after incubation in decreasing concentrations of sucrose (Fig. 3, 0 h), and some oocytes were incubated in recovery media for 1 h (Fig.3, 1 h). As shown in Fig. 3, the spindle pole machinery retains the structures at both ends of the chromosomes and a number of cytoplasmic MTOCs were also observed at 0 h. In addition, both mDia2 and pericentrin were localized to these structures. mDia2 retained its position at 0 h, thereby suggesting that freezing process did not affect the spindle pole localization of mDia2. Alternatively, mDia2 may have rapidly localized to the spindle pole within several minutes of warming.

### Growing microtubules near mDia2 in thawed oocytes

**Figure 4:**
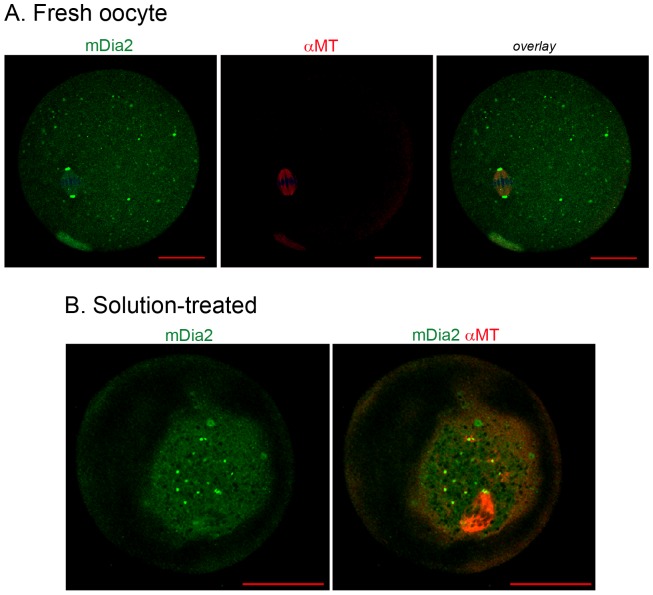
Co-staining of mDia2 and tubulin in MII oocytes and vitrification solution-treated oocytes. A, Immunofluorescence staining of mDia2 and tubulin in fresh MII oocytes. mDia2, green; α-tubulin, red. B, Immunofluorescence staining of mDia2 and tubulin in oocytes treated with equilibration and vitrification solutions. Note the shrinkage of the ooplasm because of osmosis. mDia2, green; α-tubulin, red.

**Figure 5:**
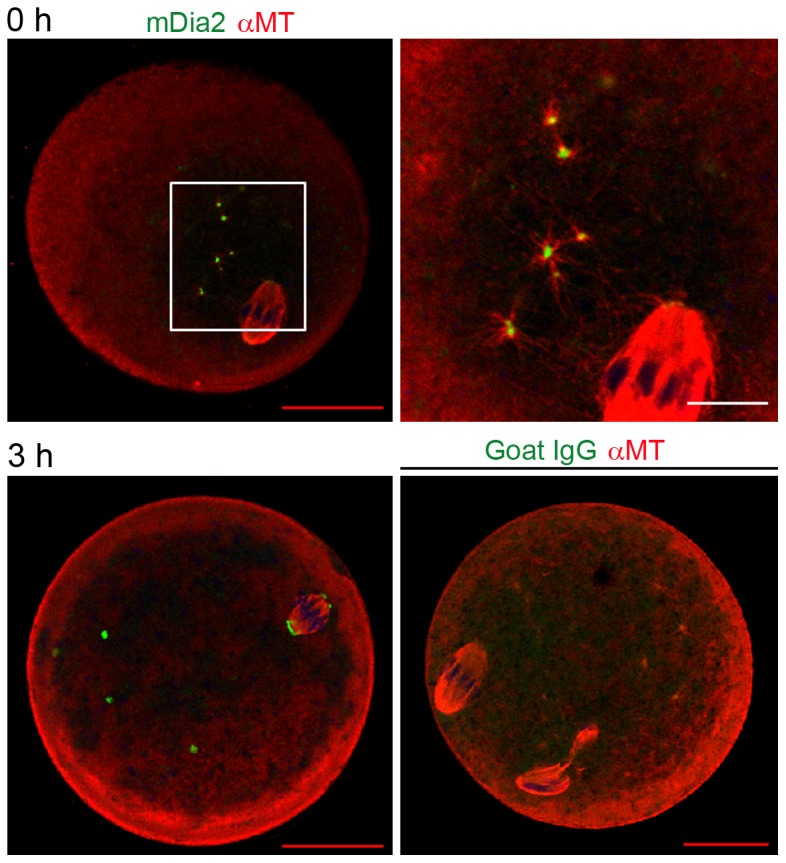
Localization of mDia2 and tubulin at the time of thawing. Vitrified MII oocytes were stored in LN_2_ for 2 weeks. Oocytes were taken out from LN_2_, incubated in decreasing concentrations of sucrose, and then fixed immediately (0 h). Some oocytes were incubated in 37 C for recovery for 3 h after thawing (3 h). These oocytes were subjected to immunofluorescence staining with anti-mDia2 and anti-α-tubulin antibodies. Green, mDia2; red, α-tubulin. In the oocyte at 0 h, mDia2 localization at spindle poles is not visible because the image is focused to MTOCs with emanating microtubules. Goat IgG was used instead of anti-mDia2 antibody in double immunofluorescence staining as a negative control (bottom right).

Next, we examined mDia2 localization and its association with themicrotubules. Double immunofluorescence staining using anti-mDia2 and anti-α-tubulin was performed (Fig. 4 and Fig. 5). In fresh MII oocytes, mDia2 was identified at each end of the meiotic spindle, as expected. To examine if the CPA-containing equilibration and vitrification solutions affect spindle morphology, MII oocytes were exposed to these solutions only and were not vitrified. As shown in Fig. 4B, CPA caused shrinkage of the ooplasm, but the spindle and mDia2-decorated spindle poles retained their morphology. mDia2 was shown to directly bind and stabilize microtubules in vitro [30], [31]. Therefore, we examined the time-based association of mDia2 with microtubules during thawing and recovery. Immediate fixation of the vitrified oocytes after incubation in decreasing concentrations of sucrose (Fig. 5, 0 h) showed microtubules emanating from mDia2 clusters in the ooplasm. This was prominent in the oocytes at 0 h, but was not visible in the oocytes after recovery (3 h). In all experiments, goat IgG was used as a negative control and it did not generate any specific signal (Fig. 5).

## Discussion

The mouse Diaphanous family of genes, which is a subfamily of formin genes, are the mammalian homologs of Drosophila diaphanous [32]. Three mDia genes, namely mDia1, mDia2, and mDia3 are known, and they share the conserved formin homology (FH) domains as well as the N-terminal Rho-binding domain. In this study, we show that mDia2 is a component of MTOCs and spindle poles of mouse oocytes, and that mDia2 is closely associated with pericentrin before and after vitrification and thawing. We previously showed that mDia2 co-localizes with γ-tubulin in both cultured cells and mouse oocytes [15]. This was the first report to suggest a role for mDia2 in spindle pole dynamics. We herein show the presence of growing arrays of microtubules from mDia2 clusters within the ooplasm (Fig. 5). MTOCs in mammalian oocytes closely resemble centrosomes functionally, and they share many centrosomal components, such as pericentrin and γ-tubulin [10], [13], [14]. mDia2 co-localizes with both of these proteins, thereby suggesting that mDia2 is a novel component of MTOCs in mouse oocytes. Members of the mDia family show differential preference toward small GTPase-binding proteins [33]. Several small GTPase-binding proteins, including Cdc42 and Rac, are shown to be important for meiotic maturation of oocytes [11]. Further investigation is required to determine whether the distribution and function of mDia2 in mouse oocytes is modulated by small GTPases.

Several studies have shown that a member of the formin protein, Fmn2, is crucial for spindle migration [2]–[4]. Fmn2 nucleates actin cables necessary for spindle migration during metaphase I, which is a mechanism of symmetry breaking in oocytes [2]. In mouse oocytes, mDia1 exhibits a specific localization pattern on meiotic spindles, and this pattern is similar to Fmn2 localization [15]. A recent report suggested that another formin protein, Daam1, is expressed in mouse oocytes and that its expression is modulated by microRNA [34]. In conjunction with our finding that mDia2 is a novel component of spindle poles, it appears that several formin proteins are involved in the maturation process of mouse oocytes. However, further investigation is required to determine whether the expression and function of various formin proteins in oocytes are conserved in oocytes of other mammalian species.

In regard to the quality assessment of cryopreserved oocytes, the location, angle, and length of spindle have previously been the main parameters of scrutiny [21], [35], [36]. Polscope enables the visualization of spindles in oocytes without fixation [22], [26], [37], but it is difficult to assess spindle pole structures by this method due to relatively low image resolution. Recovery of any loss of cellular microtubules and meiotic spindle due to cold injury would involve MTOCs, because microtubules grow from these structures in oocytes [10]. Spindle recovery at warming was shown to be faster in vitrified human oocytes than in slow-frozen oocytes [27]. In our experimental system, vitrified oocytes underwent ∼10 min of incubation in decreasing concentrations of sucrose at room temperature and then were fixed immediately (shown as 0 h in Fig. 3&5). At this time point, the meiotic spindle and mDia2-decorated spindle poles were clearly visible by immunofluorescence staining (Fig. 3). Thus, the main body of the meiotic spindle may not experience total disassembly during freezing, and if it were disassembled, it rapidly recovers within a few minutes at thawing. Our method of imaging the spindle pole by using mDia2 immunofluorescence staining enables the spindle structure and pole formation to be scrutinized. An interesting observation was that new arrays of microtubules were shown to grow from mDia2-enriched MTOCs at 0 h (Fig. 5). Therefore, some cellular microtubules are newly assembled at thawing and this process may be associated with the microtubule-induced recovery of cytoplasmic volume and re-distribution of subcellular organelles [38]. In conclusion, our work suggests that mDia2 is a novel tool for the assessment of spindle pole dynamics in cryopreserved oocytes.

## Materials and Methods

### Materials

The antibodies used were goat polyclonal anti-mDia2 (M16, Santa Cruz Biotechnology, Santa Cruz, CA, USA), mouse anti-pericentrin (#61814, BD Biosciences, San Jose, CA, USA), and mouse monoclonal anti-α-tubulin (clone DM1A, Sigma-Aldrich, St. Louis, MO, USA) antibodies. The secondary antibodies used were chicken anti-goat IgG-Alexa Fluor 488 (Life Technologies, Carlsbad, CA, USA) and donkey anti-mouse IgG-Alexa Fluor 568 (Life Technologies).

### Oocyte collection

Mice were maintained in accordance with the policies of the Konkuk University Institutional Animal Care and Use Committee (IACUC). The study conducted herein was approved by IACUC (approval number KU12081). The mice were kept in a controlled barrier facility in Konkuk University. Four-week-old ICR mice (Orient-Bio, Kyunggi-do, Korea) were injected with 5 IU of pregnant mare's serum gonadotropin (Sigma-Aldrich) to induce folliculogenesis. At 48 h post-injection, the mice received 5 IU human chorionic gonadotropin (hCG). Ovulated cumulus-oocyte complexes (COCs) were collected by oviduct flushing 13–14 h post-hCG. The cumulus cells were removed by treating the COCs with hyaluronidase (300 µg/ml, Sigma-Aldrich) for 2 min. Metaphase II(MII) oocytes were collected and transferred to Quinn's Advantage® medium containing HEPES (Cooper Surgical, Trumbull, CT, USA) and 20% fetal bovine serum (FBS, Sigma-Aldrich) at 37°C.

### Vitrification and warming of mouse oocytes

The vitrification solutions contained ethylene glycol (EG, Sigma-Aldrich) and dimethyl sulfoxide (DMSO, Sigma-Aldrich) as cryoprotectants [35], [39]. The oocytes were first pre-equilibrated in an equilibration solution containing 7.5% EG, 7.5% DMSO, and 0.5 M sucrose (Fisher Scientific, St. Louis, MO, USA) for 2.5 min at room temperature [39]. The oocytes were then transferred to the vitrification solution containing 15% EG, 15% DMSO, and 0.5 M sucrose. After 20 sec, the oocytes were loaded onto Cryotop strips (Kitazato Corporation, Shizuoka, Japan) and kept in liquid nitrogen (LN_2_) for 2–4 weeks [40]. For warming, the Cryptop strip was taken out from LN_2_ and were directly placed in the thawing media (0.5 M sucrose and 20% FBS in PBS) for 2.5 min. Thawed oocytes were collected and sequentially incubated in solutions containing decreasing concentrations of sucrose (0.25 M, 0.125 M, and 0 M) for 2.5 min each. Some oocytes were fixed at this point (0 h, Figs. 3 and 5). Finally, the oocytes were transferred to Quinn's-HEPES media containing 20% FBS and were incubated at 37°C in 5% CO_2_ for recovery (1 or 3 h). The oocytes without noticeable morphological deformation or discoloration were selected and used for further experiments. More than 90% of the oocytes survived after the thawing and were subjected to further analysis.

### Immunofluorescence staining and confocal microscopy

Immunofluorescence staining of the oocytes was performed using a drop culture system. The oocytes were fixed in 4% paraformaldehyde and 0.1% Triton X-100 in PBS for 30 min. After washing in 0.1% Triton X-100 in PBS, the oocytes were blocked in 2% bovine serum albumin (BSA) in PBS for 90 min. The oocytes were incubated with the primary antibody (2 µg/ml) in 2%BSA/PBS at 4°C overnight. The next morning, the oocytes were washed three times in 2% BSA/PBS and incubated with the secondary antibody in 2% BSA/PBS. After three washes in 2% BSA/PBS, the oocytes were stained with TO-PRO-3-iodide (1∶250, Life Technologies). After a final wash in 2% BSA/PBS, the oocytes were directly placed onto a glass slide and covered with a glass coverslip. As a mock control, goat IgG was used at each staining. Images were obtained using the Olympus Fluoview™ FV1000 Confocal Microscope (Tokyo, Japan) equipped with the multi Argon-ion (457, 488, and 515 nm), He-Ne (green, 543 nm), and He-Ne (red, 633 nm) lasers. The images were analyzed using the software Fluoview version 1.5, a platform associated with the confocal microscope.
